# Cloacal Swabs Are Unreliable Sources for Estimating Lower Gastro-Intestinal Tract Microbiota Membership and Structure in Broiler Chickens

**DOI:** 10.3390/microorganisms8050718

**Published:** 2020-05-12

**Authors:** Travis Williams, Giridhar Athrey

**Affiliations:** 1 Department of Poultry Science, Texas A&M University, 101 Kleberg Center, 2472 TAMU, College Station, TX 77843-2472, USA; twilliams49@tamu.edu; 2 Faculty of Ecology and Evolutionary Biology, Texas A&M University, 101 Kleberg Center, 2472 TAMU, College Station, TX 77843-2472, USA

**Keywords:** microbiota, chicken gut microbiota, broiler, cloacal swabs, 16S rRNA, cecal microbiota, next generation sequencing, diagnostic test method, avian

## Abstract

The gastrointestinal microbiota of chickens plays a central role in health and performance. Cloacal swabs, due to their proximity to the ceca (a vital site of functional activity), are an alternative, non-invasive method used for assaying microbial communities and might be a viable option for longitudinal studies. In this study, the microbiota of twenty paired cecal content and cloacal swab samples representing two dietary treatments was assessed using 16S rRNA V4 hypervariable region sequencing and was analyzed using the MOTHUR pipeline, Phyloseq, and Vegan packages. Paired t-test and Wilcoxon signed-rank tests showed significant differences in the Chao1 index (*p*-value <0.0001 and *p*-value <0.0001, respectively) but not in the Inverse Simpson species diversity estimator (*p*-value = 0.06763 and *p*-value = 0.06021, respectively) between the cecal content and cloacal swabs. β-diversity between the cloacal swabs and cecal microbiota also showed significant differences using PERMANOVA, HOMOVA, and weighted UniFrac testing (*p*-values < 0.001). Based on a paired sample analysis, this study provided evidence of the high inter-individual variation and randomness of cloacal microbiota, in contrast to cecal microbiota. Our findings indicated that cloacal swabs do not approximate the α or β diversity of cecal samples and are not suitable for longitudinal studies of gut microbiota.

## 1. Introduction

Chicken (*Gallus gallus domesticus*) is the source of the most consumed animal protein globally, at nearly twice the amount of pork and beef combined [1]. Due to the demands on production, there is a great emphasis on improving poultry health and performance [2,3,4,5]. Notably, the role of gut microbiota in improving performance [6,7,8], welfare [9], and health [4,10,11,12,13,14], is a topic of intense interest. The gut microbiota is studied intensively in chicken; an NCBI PubMed Central search for “Poultry Gut Microbiota” yielded 2586 research articles within the last five years.

The gut microbiota, an ecological community of commensal and non-commensal microorganisms [15,16], is found throughout the entire length of the broiler′s gastrointestinal tract (GIT), although most research concentrates on the organs within the lower sections—the small intestine (duodenum, jejunum, and ileum), large intestine, cecum, and cloaca. The ceca, a pair of blind sacs, are especially important as the site of functional activity relevant to microbial communities and species studied in performance and health [7,17]. The ceca retain nearly 10^11^ microbial cells per gram and are an important location for fluid resorption via the translocation of urea from the urodeum and the fermentation of carbohydrates [17,18,19,20,21]. As a consequence, the ceca are the most sampled gut segment in chicken gut microbiota studies [7,22]. A standard experimental method of microbiota analysis in poultry involves the invasive sampling of the ceca, following euthanasia, which prevents longitudinal studies of the same experimental animals. 

Cloacal (or vent) swabs are an alternative, non-invasive method used on domestic, migratory, or endangered bird species [23], where invasive sampling might not be permitted. Due to the non-invasive aspect, cloacal swabs are frequently used for assaying and monitoring agents, such as *Salmonella* spp [23], avian influenza [24,25,26], coccidiosis [27], and *Campylobacter coli* [28]. Importantly, these swabs were analyzed using real-time polymerase chain reaction (qPCR) or microorganism-specific plating methods and not for the total microbiota analysis. 

Due to the ubiquity of cloacal swabbing, mainly for diagnostics, it is critical to determine if and how representative cloacal microbiota are of cecal microbiota. Cecal microbiota in chicken show broad similarities with lower large-intestinal microbiota [29], and the cloaca abuts the large-intestine. If cloacal microbiotas approximate the cecal microbiota, it would enable non-invasive longitudinal studies. On the other hand, if cloacal microbiota is not a reliable proxy for cecal microbiota occurrence and abundance, then its utility for assessing avian microbiota would be limited. This relation has been investigated previously to some extent by Kers et al. [29,30]. While their 2019 [30] investigation compared the α and β diversity of cloacal swabs to other lower GIT tract microbiota, the focus of this study was on the overall effects of broiler age, farm location, and sampling method on microbial diversity. Our study, in contrast, focuses directly on the resolution and repeatability of microbiota patterns. To resolve the reciprocity of cecal and cloacal microbiotas, we used a paired-sample approach to compare cecal and cloacal microbiota communities sampled from the same individuals. Based on previously published works on fecal microbiota, we hypothesized that cloacal microbiota is not representative of cecal microbiota from the same individuals. We used 16S rRNA sequence-based analysis of α and β diversity of the communities, between the two sampling methods. Here, we report that cloacal swabs are unreliable representatives of the presence–absence of taxa, as well as α and β diversity.

## 2. Materials and Methods 

### 2.1. Study Design

Twenty, fast-growth, high-yield commercial broilers were randomly sampled from a study conducted at the Texas A&M University Poultry Science Research Center in College Station, TX, USA. Samples taken represent two dietary treatments with two replicate pens, each pen containing five broilers, whose ages were thirty-three to thirty-six days of age. We raised broilers under standard industry lighting conditions with ad libitum feed and water. The broilers in this study were raised on two diet treatments. Treatment 1 (T1) consisted of a corn, soybean meal protein (35%) diet, whereas Treatment 2 (T2) consisted of a corn-based diet, with mixed protein sources (bone meal, corn gluten meal, fish meal, and soybean meal). Both diets were energetically equivalent. For each diet treatment, we raised birds in replicate pens. We included diet treatments as a factor to assess the ability of cecal versus cloacal swabs to differentiate between dietary treatments. 

### 2.2. Sample Collection

From each dietary treatment (T1, T2), we randomly sampled ten broilers. Within each diet treatment group, an equal number of individuals were sampled (five). We transported the randomly selected birds to a clean room for cloacal swab collection, euthanasia, and post-mortem sample collection from the ceca. For cloacal swabbing, we used a Puritan PurFlock Ultra Sterile mini-tip Flock swab with a sterile container (Puritan, Guilford, ME, USA) to sample the cloacal microbiota from live birds, following a modified protocol originally reported by Vo & Jedlicka [31]. First, the exterior surface of the cloaca was wiped with a cotton ball sprayed with 70% Ethanol. The PurFlock swab was gently inserted approximately 22 mm into the cloaca, a depth just beyond the length of the swab tip. The swab was rotated five times in a slow clockwise motion around the cloaca, applying moderate pressure so that the swab-tip maintained contact. Additionally, we rolled the swab-tip so that the entire surface of the swab was coated with cloacal material. Following sample collection, the swab was inserted into the supplied sterile container, immediately placed on ice after collection, and transferred to a –80 °C, freezer until further processing.

After completing the cloacal swab sample collection, individual broilers were euthanized by carbon dioxide (CO_2_) exposure, followed by cervical dislocation. All animal procedures were reviewed and approved by the Texas A&M University Institutional Animal Care and Use Committee (IACUC). This study was performed using approved animal use protocol, IACUC 2016-0064. We used sterile instruments for the post-mortem sample collection from the cecal content. We collected cecal content samples within thirty minutes of euthanizing the broiler. Approximately 2 g of the cecal content material was collected and immediately stored in a 1:5 ratio (w/v) of RNAlater (QIAGEN, Hilden, Germany). Tissue samples were stored at 4 °C for twenty-four hours (following the RNAlater storage protocol) and then moved to a −80 °C freezer, until further processing.

### 2.3. Sample Preparation and Nucleic Acid Isolation 

Total genomic DNA (gDNA) was isolated from approximately 0.1 g of cecal content material using a QIAmp PowerFecal DNA Kit, following the manufacturer’s protocol (QIAGEN, Hilden, Germany). Briefly, this protocol followed a bead-beating sample homogenization step in supplied bead tubes, followed by column-based extraction and elution. gDNA from the cloacal swabs was isolated following a modified extraction protocol using the DNAzol Reagent (Invitrogen, ThermoFisher Scientific, Waltham, MA, USA). The modified protocol was as follows. First, we suspended the swab-tip in a 406 μL solution containing 1X tris buffered saline (TBS), 0.01 M ethylenediaminetetraacetic acid (EDTA), and 0.005% (w/v) Tween-20 (Sigma-Aldrich Corp, St. Louis, MO, USA) to release the material from the swab-tip. The suspension, along with the trimmed swab tip and 1.7 g of 1 mm zirconia beads (BioSpec Products, Bartlesville, OK, USA), was placed in screw cap tubes and vortexed for ten seconds. Following vortexing, 1 mL of DNAzol was added to the swab-tip solution and homogenized for sixty seconds at 1500 rotations per minute (rpm), using a BioSpec Mini-Beadbeater-96 machine (BioSpec Products, Bartlesville, OK, USA). The homogenized solution was left to rest for an additional ten minutes at room temperature, to allow the DNAzol to lyse the phospholipid bilayer of the prokaryotes suspended in the solution. The DNA pellet was further purified and precipitated using 3 M sodium acetate and 100% ethanol. The purified pellet was washed with 70% ethanol a second time, to remove any polymerase chain reaction (PCR) inhibitors or residual chemicals left on the pellet. We suspended the DNA pellet in 100 μL to 450 μL of 8 mM sodium hydroxide (NaOH) and 2.3 μL 1 M HEPES per 100 μL of NaOH, for storage. Final gDNA concentrations and purity of all samples were measured on a NanoDrop n1000 (ThermoFisher Scientific, Waltham, MA, USA) before 16S rRNA PCR amplification. 

While it is known that different extraction methods can yield different quantities of dsDNA, the effect of the extraction method on the microbiota detected is considered to be marginal, which is determined mostly by sample type [32]. Therefore, we did not expect the microbiota differences to be driven by the DNA isolation methods.

### 2.4. PCR, Library Preparation, and Sequencing for 16S rRNA Analysis

To amplify the 16S rRNA V4 hypervariable region, we performed a 50 μL PCR reaction consisting of 10 μL of template DNA (150 ng to 185 ng total), 13.8 μL nuclease-free water, 0.6 μL (25 μM) forward primer (Hyb515F_rRNA: 5′-TCGTCGGCAGCGTCAGATGTGTATAAGAGACAGGTGYCAGCMGCCGCGGTAA–3′), 0.6 μL (25 μM) reverse primers (Hyb806R-rRNA: 3′- TAATCTWTGGGVHCATCAGGGACAGAGAATATGTGTAGAGGCTCGGGTGCTCTG-5′) [14,33], and 25 μL of NEBNext^®^ High-fidelity 2X PCR Mastermix containing the hot-start Q5^®^ High-Fidelity DNA polymerase (New England BioLabs, Ipswich, MA, USA). Sequences of forward and reverse primers contain Illumina adapters, primer pad, and primer linker. We performed PCRs in triplicates (per sample) on an Eppendorf Mastercycler Pro (Eppendorf, Hamburg, Germany), with the following cycle conditions—initial single cycle denaturation step at 98 °C for 30 s, twenty-five cycles at 98 °C for 10 s, 65 °C for 30 s, and 72 °C for 30 s, with a single cycle final extension step at 72 °C for 5 min. A non-template negative control (nuclease-free water used to prepare all solutions) and positive template control (cultured *E. coli*) were included in all PCR amplification reactions. All amplicon products were visualized on a 2% agarose gel. No amplification bands were observed in the negative controls and were, therefore, not included in library preparation and sequencing. Before sequencing, amplicon products containing Illumina barcodes were cleaned using Ampure Magnetic beads (Beckman Coulter, Brea, CA, USA). Amplicon pools were barcoded and sequenced at the Genome Sequencing and Analysis Facility (GSAF, University of Texas, Austin, TX, USA). We sequenced the libraries on an Illumina MiSeq platform (Illumina, San Diego, CA, USA), running in 250 base pair paired-end mode.

### 2.5. Bioinformatic Pipeline for Microbiota Evaluation

Resultant fastq files from sequencing were processed using the MOTHUR software v. 1.39.5 [34]. Briefly, paired-end reads were joined using the make.contigs command. We aligned the sequences to the SILVA database v. 132 [35] and removed the chimeric sequences using the UCHIME program v. 4.2.40 [36]. Low abundance operational taxonomical units (OTU′s) and singletons were removed from analysis with the split.abund command, using a cutoff = 1. Chloroplast, Mitochondria, Eukaryota, and other unknown sequences were removed from the dataset using the remove.lineage command. Total OTU’s were then generated at the species level (0.03) and then classified using the classify.otu and dist.seqs commands, respectively. OTU tables and other output from MOTHUR were further analyzed on the R platform v. 3.6.2 [37] using the Phyloseq v. 1.28.0 [38] and Vegan v. 2.5-6 [39,40] packages. We generated plots using the ggplot2 package v. 3.3.0 [41]. 

We performed principal coordinates analyses (PCoA) based on the Bray–Curtis distance using the Phyloseq and ggplot2 packages. Rarefaction curves summary statistics (Figure S1A and Figure S1B) and Q–Q plots of the Good′s coverage index values (Figure S2A and Figure S2B) are presented in the supplementary materials. We compared family level microbial composition using the relative abundance values, after removing the low abundance taxa (<2%).

To assess whether the abundance structures (ignoring taxonomic composition) between the cloacal and cecal communities were comparable, we performed non-parametric tests on the relative abundance and cumulative distribution functions of the paired cecal and cloacal swab datasets. The Q–Q plots were generated for each comparison to check for normality. We used the two-sample Kolmogorov–Smirnov (KS) test using the ks.test function in R {base} to assess whether the microbiota populations (cecal vs. swabs) are from the same distribution. The two-sample ranked location-scale tests of Cucconi and Lepage were implemented using the nonpar package v. 0.1-2 [42] using the cucconi.test and lepage.test commands, respectively. The Cucconi test is a ranked test that assesses whether the locations and scales of the two population distributions are equal [43,44,45], while the Lepage test is a ranked location-scale test that combines the Ansari–Bradley test for scale and the Wilcoxon–Mann–Whitney test for location [46,47].

### 2.6. Statistical Tests for α and β Diversity

Two statistical tests were performed in R to evaluate the α and β diversity between sampling locations and amongst dietary treatments within each sampling location. We used the two-sample Wilcoxon signed-rank test to compare the Chao1 and Inverse Simpson (InvSimpson) α diversity measures. To compare β diversity, we used the permutational multivariate analysis of variance (PERMANOVA) using the “Adonis” function of the Vegan package with 9999 permutations [48,49]. In addition to PERMANOVA, we compared β diversity in MOTHUR using HOMOVA, AMOVA, and unifrac.weighted [50]. We applied the weighted UniFrac test to investigate the probability that two or more communities have the same structure by chance. These three species-level non-parametric tests were computed using the Yule and Clayton measure of dissimilarity average phylogenetic distances [51]. The statistical significance of all comparisons was assessed at *α* = 0.05.

## 3. Results

### 3.1. Sampling Location Yields a Variability in Sequencing Depth 

The raw data from sequencing generated a total of 694,559 reads, with an average of 18,278 reads per sample. Total read depth per sample was limited to an arbitrary minimum of 3005 to ensure adequate read depth in any given sample [52], and thus was the cutoff for inclusion in further analysis. One cloacal swab library out of the 40 total libraries sequenced was excluded as a result of this read threshold and to retain the paired nature of our analysis, the corresponding cecal content sample was excluded. We proceeded with nineteen paired samples for which both the cloacal swab and the cecal data was found. A summary of the complete information can be found in Table S1 and Table S2, respectively. The cecal samples had an average of 22,656 reads (IQR 20,312–24,515 reads), whereas the cloacal swabs had an average of 13,900 reads (IQR 6902–20,366 reads). The average Good’s coverage for all thirty-eight samples was 99.57%, (standard deviation = 0.305%), showing that the retained datasets had adequate sequence coverage to sample OTUs. Summary statistics for both the cecal and cloacal datasets are given in Table 1.

### 3.2. Broad Differences between Cecal and Cloacal Microbiota Members

Overall, the thirty-eight samples yielded 1790 OTU’s assigned to 88 families. The top three families based on the relative abundance were *Lactobacillaceae* (11.409%, Phylum Firmicutes), *Ruminococcaceae* (9.979%, Phylum Firmicutes), and *Peptostreptococcaceae* (9.817%, Phylum Firmicutes). The nineteen cecal content samples yielded 1626 total OTU’s from 60 families with *Ruminococcaceae* (22.163%, Phylum Firmicutes), *Barnesiellaceae* (11.954%, Phylum Bacteroidetes), and *Rikenellaceae* (8.126%, Phylum Bacteroidetes) as the top three families represented, based on relative abundance. The nineteen cloacal swab samples yielded 914 total OTU′s from 82 families. The top three families in the cloacal swab samples did not show a uniform distribution. The top three most abundant families in the cloacal samples were *Lactobacillaceae* (16.588%, Phylum Firmicutes), *Peptostreptococcaceae* (10.104%, Phylum Firmicutes), and *Pasteurellaceae* (8.931%, Phylum Proteobacteria). It is noteworthy that *Peptostreptococcaceae* was only present in five out of nineteen cloacal swab samples and the family *Lactobacillaceae* were present only in six of the nine T2 cecal content samples.

**Figure 1:**
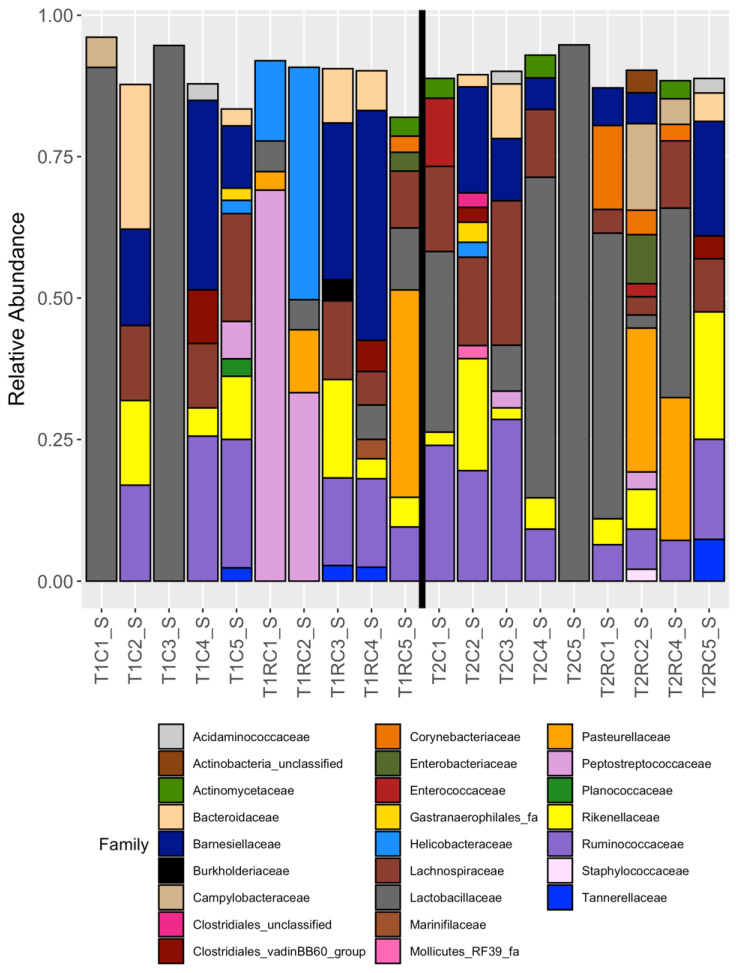
The relative abundance of families observed for each cloacal swab samples that had >= 3005 reads; *n* = 19. The vertical black line divides the samples belonging to the two dietary treatments.

**Figure 2:**
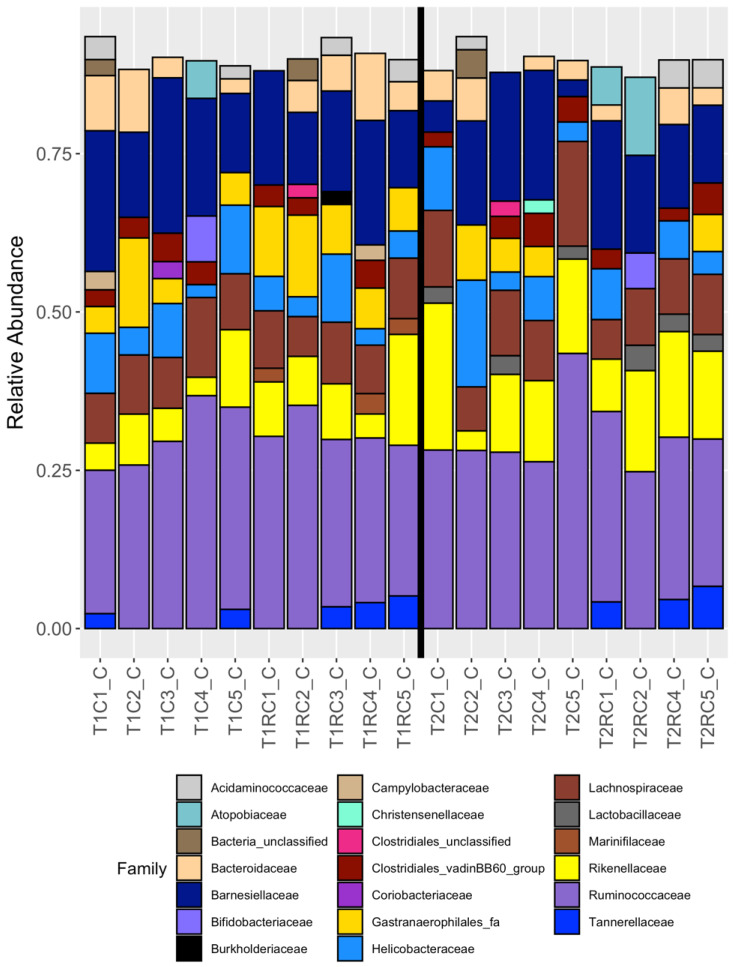
The relative abundance of families observed for each cecal content samples that had >= 3005 reads; *n* = 19. The vertical black line divides samples belonging to the two dietary treatments.

Twenty-five families were represented in the cloacal swab samples with twenty families represented in the cecal content samples, as shown in Figure 1 and Figure 2, respectively. There were eleven families that shared cecal and cloacal samples, with nine families (*Atopobiaceae*, *Bacteria_unclassified*, *Bifidobacteriaceae*, *Christensenellaceae Clostridiales_unclassified* (OTU 0087)*, Clostridiales_vadinBB60_group* (OTU 0045), *Coriobacteriaceae*, *Gastranaerophilales_fa* (OTU 0010), and *Helicobacteraceae* (OTU 0005)) unique to the cecal content and fourteen families (*Actinobacteria_unclassified, Actinomycetaceae*, *Clostridiales_unclassified* (OTU 0096), *Clostridiales_vadinBB60_group* (OTU 0037)*, Corynebacteriaceae*, *Enterobacteriaceae*, *Enterococcaceae*, *Gastranaerophilales_fa* (OTU 0014)*, Helicobacteraceae* (OTU 0021)*, Mollicutes_RF39_fa*, *Pasteurellaceae, Peptostreptococcaceae*, *Planococcaceae, and Staphylococcaceae*) unique to the cloacal swabs.

**Figure 3:**
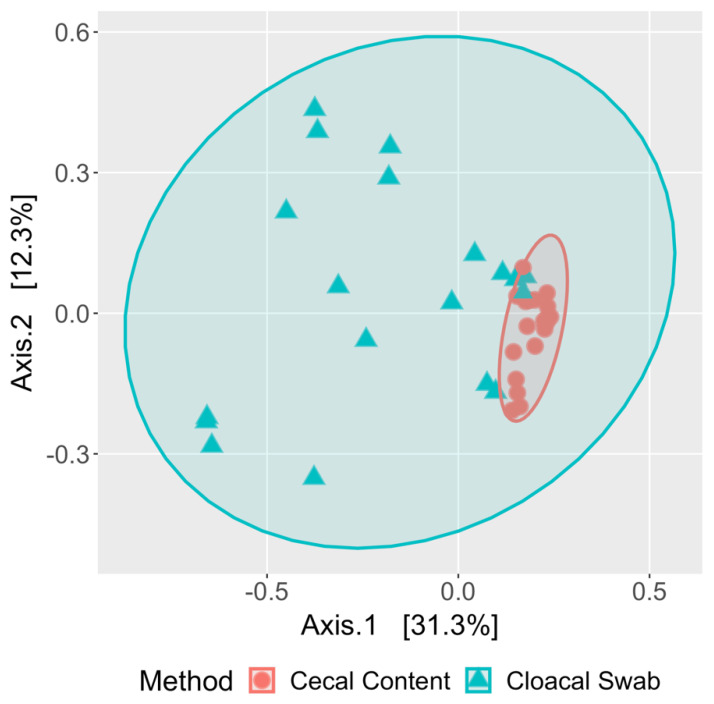
Principal component analysis (PCoA) comparing the cecal content and the cloacal swab testing methods. PCoA plot is based on the Bray–Curtis distances and showed that the cecal samples cluster tightly together, while the cloacal swab samples showed high variability. The ellipses represent the 95% confidence intervals for each sample group.

Based on the PCoA analysis comparing cecal and cloacal samples, the cecal content samples clustered tightly together, whereas the cloacal swab samples showed high variability and limited overlap with the cecal content samples (Figure 3). This high variability was not surprising, given the total number of families represented in the cloacal swabs. Overall, the ordination pattern of these paired samples showed broad-ranging differences between the two sampling approaches.

The cumulative distribution functions of the relative abundances between the cecal and cloacal microbiotas were also significantly different. The KS (D = 0.11995, *p*-value = < 0.0001) and Cucconi tests (C = 426.916, *p*-value < 0.0001) were highly significant. The results of the Kolmogorov-Smirnoff and Cucconi tests on the cumulative distribution function further demonstrated that both the location and the scales of the cecal content and cloacal swab distributions of relative abundances were highly different.

### 3.3. Richness and Diversity Differences between the Cecal and Cloacal Samples 

**Figure 4:**
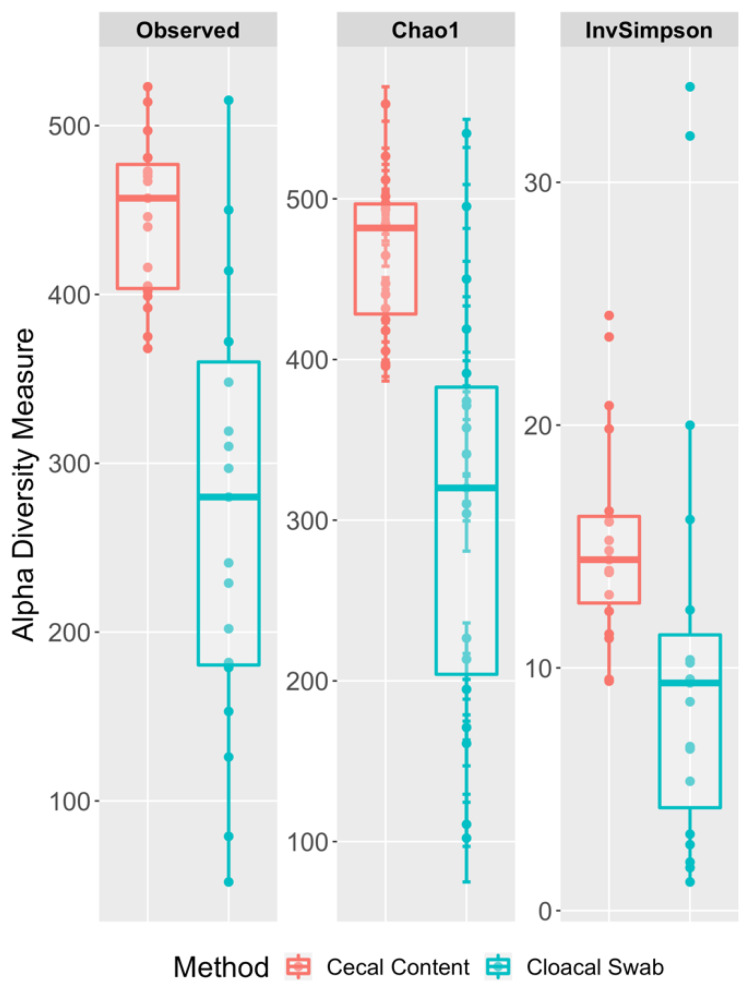
Boxplots of the observed, Chao1, and Inverse Simpson (InvSimp) α diversity indices for the comparison of cecal content and cloacal swab samples.

We compared microbial species richness and diversity of the cecal content and cloacal swabs using the Chao1 and Inverse Simpson estimators (Figure 4). Both the Wilcoxon signed-rank test (W = 4, *p*-value = < 0.0001) and paired t-test (t = 5.7938, *p*-value < 0.0001) showed highly significant differences in the Chao1 index between the cecal content and the cloacal swabs, with the highest richness observed in the cecal samples. A higher Chao1 value indicated a higher number of low abundance taxa, e.g., singletons [53,54]. The higher value in cecal samples, suggests that the rarer taxa were captured in the cecal samples. However, the Inverse Simpson species diversity estimator was not different between the cloacal and cecal samples based on a Wilcoxon signed-rank test (W = 48, *p*-value = 0.06021) and a paired t-test (t = 1.9445, *p*-value = 0.06763). Similar to the Chao1 findings, the cecal content had higher microbial diversity, compared to the cloacal swabs. As the Inverse Simpson index estimates the richness weighted by the proportional abundance of taxa present within the samples, the non-significance suggests that the two types did not differ in their internal weighted abundances.

**Figure 5:**
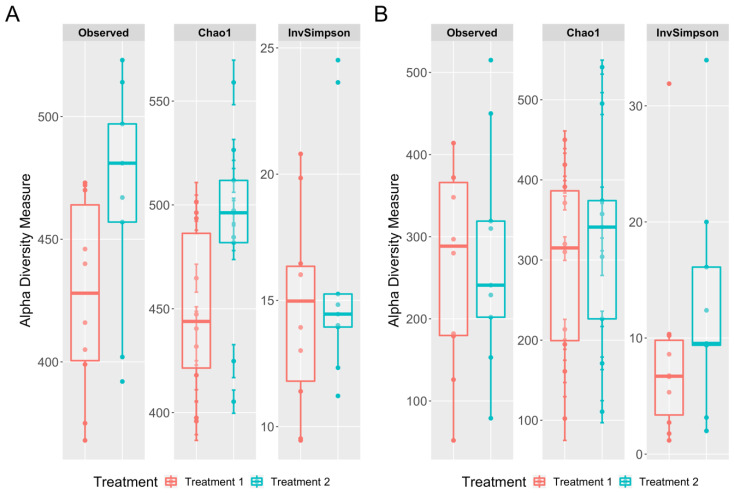
(**A**) Boxplot of the observed, Chao1, and Inverse Simpson (InvSimpson) measurements for the alpha diversity of the cecal content samples by treatment. (**B**) Boxplot of the observed, Chao1, and Inverse Simpson (InvSimpson) measurements for the alpha diversity of the cloacal swab samples by treatment.

To assess whether cecal or cloacal swabs captured differences between dietary treatments, we performed richness and diversity analyses, comparing the two diets. Figure 5A shows the comparisons within cecal data, and Figure 5B shows the cloacal swab comparisons. The Wilcoxon signed-rank test on the Chao1 estimator returned non-significant *p*-values for the cecal content treatments (W = 24, *p*-value = 0.09472) and the cloacal swab treatments (W = 40, *p*-value = 0.7197). The Chao1 values were higher for T2 than T1 in both the cecal content and the cloacal swab methods. Similarly, the Inverse Simpson index was not different between the diets based on the cecal samples (W = 40, *p*-value = 0.7197) or the cloacal swab data (W = 27, *p*-value = 0.1564). In summary, neither the cecal nor the cloacal swab samples showed significant differences between the dietary treatments based on the Chao1 and Inverse Simpson indices.

### 3.4. Cloacal Swabs do not Reflect the Community Structure Inferred from Cecal Samples

The PERMANOVA analysis showed a significant difference (F. Model = 8.3319, R^2^ = 0.18794, *p*-value = 0.0001) in the centroids and dispersion between the cecal and cloacal microbiota. This difference of community structure was further supported with significant results from the HOMOVA (BValue = 3.6193, *p*-value < 0.001) and the weighted UniFrac (WScore = 0.750238, WSig < 0.001) tests. On the whole, the results from the PERMANOVA, HOMOVA, and the weighted UniFrac tests all showed that the microbiota communities inferred from the cloacal swabs were different from the cecal microbiota.

Next, we investigated whether differences in the diet treatments (T1 and T2) elicit differences in communities (β diversity,) inferred using cecal versus cloacal samples. We found that the cecal content sampling method detected differences in β diversity between the diets, but this difference was not observed in the cloacal swab sampling method. The results from the PERMANOVA showed that the cecal content was different between dietary treatments (F. Model = 2.1281, R^2^ = 0.11125, *p*-value = 0.0249). This observation was supported by significant HOMOVA (Bvalue = 0.806737, *p*-value = 0.024) and AMOVA (Fs = 2.6899, *p*-value = 0.044) test results. Similarly, the weighted UniFrac also returned a significant difference (WScore = 0.639013, WSig <0.001). Overall, the cecal microbiota communities were significantly different between diets. 

In contrast, all but the weighted UniFrac test returned non-significant results for the cloacal swab microbiotas. PERMANOVA (F. Model = 0.99558, R^2^ = 0.05532, *p*-value = 0.4124), HOMOVA (B value = 0.0240581, *p*-value = 0.218), and AMOVA (Fs = 1.00622, *p*-value = 0.416) all showed that the cloacal swab microbiota were not different between the two diets. In contrast, the weighted UniFrac comparison of microbiotas between diets, using the cloacal swabs was found to be significantly different (WScore = 0.620973, WSig < 0.001). 

Further comparison of the dietary treatments within and between the sampling methods using the KS, Lepage, and Cucconi tests, yielded no significant differences (Table 2). These results are surprising, considering the differences observed using the cecal samples. However, as these tests focused on the overall abundance distributions, while ignoring the taxonomic representation, these results make sense.

## 4. Discussion

In this study, we showed that the microbiota identified from the cloacal swabs are not representative of the cecal microbiota, and therefore, are not a suitable approach to sampling the microbial communities of the lower gastrointestinal tract. This result was highly surprising, given that the cecal and large intestine microbiotas are alike by week five, in chicken [29]. Not only were the cloacal communities limited in their resemblance to cecal communities, the patterns of presence–absence as inferred by the richness estimates were also significantly different. These findings suggest that there is a high degree of stochasticity to taxa sampled from the cloaca. Our results showed similarities to the findings of Videvall et al. [55], who compared cloacal swabs and fecal samples in the ostrich (*Struthio camelus*) to analyze the lower GIT microbiota community and demonstrated the inaccuracy of fecal and cloacal swabs to reflect lower GIT microbiota.

The broad-ranging differences between the cloacal and cecal microbiota mirror the patterns seen with fecal microbiota in chicken. Previous studies have demonstrated that fecal samples show qualitative similarities, but quantitative differences compared to GIT [27,56]. Stanley et al. [56] showed significant differences in the community structure of cecal and fecal samples (collected using a shallow cloacal swab). In a previous study we also reported the same pattern [14]. In this study, we used deep cloacal swabbing (approximately 22 mm depth of sampling), which is typical for diagnostic cloacal swabbing protocols [28]. Our results showed that the cloacal samples were not representative of lower GIT microbiota in birds.

### 4.1. High Variability of Cloacal Microbiota

While the factors influencing fecal microbiota differences from cecal communities (external conditions and environmental microbiota) are expected, the cloacal swab dissimilarities and variability are more surprising. It is not clear if the cloaca of chicken is colonized, unlike other parts of the GIT. While numerous surveys of cloacal microbiota exist in the literature, in wild birds, the cloacal microbiota is often the only locus for characterizing gut microbiota, as euthanasia might not be an option. However, our results showed that the taxonomic composition and community profiles obtained from the cloacal swabs can be highly random, with little consensus, even when collected under controlled conditions.

The high-interindividual differences in cloacal microbiomes were also reported for barn swallows [57]. Barn swallows have different social structures and sex-based behavioral differences, that make direct comparisons with chicken difficult, but the poor reproducibility of cloacal microbiota is, nonetheless, a notable similarity. We found lower richness and diversity of microbial taxa in the cloaca, compared to the cecal microbiota. Van Veelen et al. [58] showed lower richness and diversity of the cloacal microbiota but surmised that top-down regulation by the host′s genetics drives this pattern. However, our data showing significantly higher richness in the ceca, suggests that host genetics do not drive lower richness or diversity in the cloaca. The variability of the cloacal swab data was revealed only in contrast with the paired cecal datasets.

On the other hand, Hird et al. [59] found that cloacal microbiomes differed among the species of ducks, and by influenza infection status. In that study, the interspecies differences among ducks might have been driving the resolution of differences among the observed microbial species. Furthermore, as they did not characterize cecal microbiota, it is not possible to determine how the cloacal data compared to the cecal data. Our analysis lead us to advocate extreme caution when inferring lower GIT microbiota patterns from the cloacal swabs of birds.

Cloacal swabs are routinely used to assess infection status in domesticated, pet, and wild bird species [60,61,62]. In the majority of these cases, targeted assays (RT–PCR) use swab samples for the detection of pathogenic species. In these cases, we rely on the sensitivity of the assays to provide valuable information for treatment or containment of pathogens, especially in poultry operations. While our data showed high variability in the representation of taxa in cloacal samples, the sensitivity of the RT–PCR approaches might allow lower detection thresholds. However, the reciprocity of taxon representation with cloacal 16S rRNA sequencing and targeted PCR methods needs to be experimentally determined.

### 4.2. Resolution of Microbial Community Differences between Diets

In our analysis of microbiota between the two dietary treatments, we found that the cecal nor the cloacal samples were able to differentiate between diets, particularly α diversity. However, we emphasized that this equivalency existed aside from the fact that the cecal and cloacal communities were highly dissimilar. Additionally, while the cecal samples were similar due to the overlapping distributions between diets, the similarity of the cloacal swabs was driven by the high variability across all cloacal samples (Figure 5B). Additionally, the housing environment, rather than dietary protein source, is known to be a more significant factor driving the cecal microbiota differences. Hubert et al. [63] reported that birds raised in the same housing environment, regardless of the dietary protein source, had similar cecal microbiota. In this present study, all chickens were raised in the same barn (across replicate pens), where they were provided with the same bedding material and water source. Therefore, the high variability among cloacal samples, all collected in a controlled environment, represents, in our opinion, the high variability inherent to cloacal samples.

## 5. Conclusions

In this study, we showed that cloacal swabs do not faithfully approximate either the α and β diversity of cecal samples, based on the paired samples. Therefore, the cloacal swabs are unsuitable for assessing lower GIT microbiota in birds. While the high variability of cloacal microbiota has been reported previously, our study provided experimental evidence to capture the randomness of cloacal microbiota, in contrast to the consistency of the cecal samples. One of the consequences of our finding is that the cloacal samples, akin to fecal samples, are not suitable or reliable for longitudinal studies of gut microbiota patterns in birds. Finally, the high inter-individual variation of cloacal swab data warrants an experimental assessment of their reliability for targeted diagnostic methods.

## Figures and Tables

**Table 1:** Descriptive statistics of the sequencing data for the nineteen cecal content and nineteen cloacal swabs.

Descriptive Statistic	Cecal Content Reads	Cecal Content Good’s Coverage	Cloacal Swab Reads	Cloacal Swab Good’s Coverage
Sample Size	19	19	19	19
Minimum	18,106	99.61%	3005	98.70%
1st Quartile	20,312	99.70%	6902	99.15%
Median	21,662	99.72%	11,043	99.49%
Mean	22,656	99.72%	13,900	99.41%
3rd Quartile	24,515	99.74%	20,366	99.72%
Maximum	29,088	99.86%	31,862	99.86%
IQR	4204	0.043%	13,464	0.576%
Range	10,982	0.25%	28,857	1.16%

**Table 2:** Summary of the statistical test used in comparing the geometric mean distributions of dietary treatments within methods. Dietary comparisons are listed in the first column, with the statistical tests for the specified comparison in the adjacent columns. Each cell contains the respective test statistic and *p*-value for that comparison.

Comparison	KS Test	Lepage Test	Cucconi Test
Cecal Content T1 vs. Cecal Content T2	D = 0.2569; *p*-value = 0.5494	L = 1.1637; *p*-value = 0.5625	C = 0.346; *p*-value = 0.705
Cloacal Swab T1 vs. Cloacal Swab T2	D = 0.1773; *p*-value = 0.8199	L = 1.853; *p*-value = 0.4033	C = 0.733; *p*-value = 0.523

## Supplementary Materials

The following are available online at https://www.mdpi.com/2076-2607/8/5/718/s1. Figure S1: Rarefaction curves for the nineteen cloacal swab samples and the nineteen cecal content samples. Figure S2: Q–Q plots for the cloacal swabs and the cecal content. Table S1: Raw sequencing data summaries for the nineteen cloacal swab samples. Table S2: Raw sequencing data summaries for the nineteen cecal content samples.
