# Supplementary material for: Cloacal Swabs Are Unreliable Sources for Estimating Lower Gastro-Intestinal Tract Microbiota Membership and Structure in Broiler Chickens
Source: Microorganisms. 2020 May 12;8(5):718. doi: 10.3390/microorganisms8050718 (PMC7285018; doi:10.3390/microorganisms8050718)
Supplement: Supplementary file 1 [file microorganisms-08-00718-s001.pdf]

microorganisms

MDPI

Supplemental Materials

Figure S1. Rarefaction curves for the nineteen Cloacal Swab samples and the nineteen Cecal Content samples.

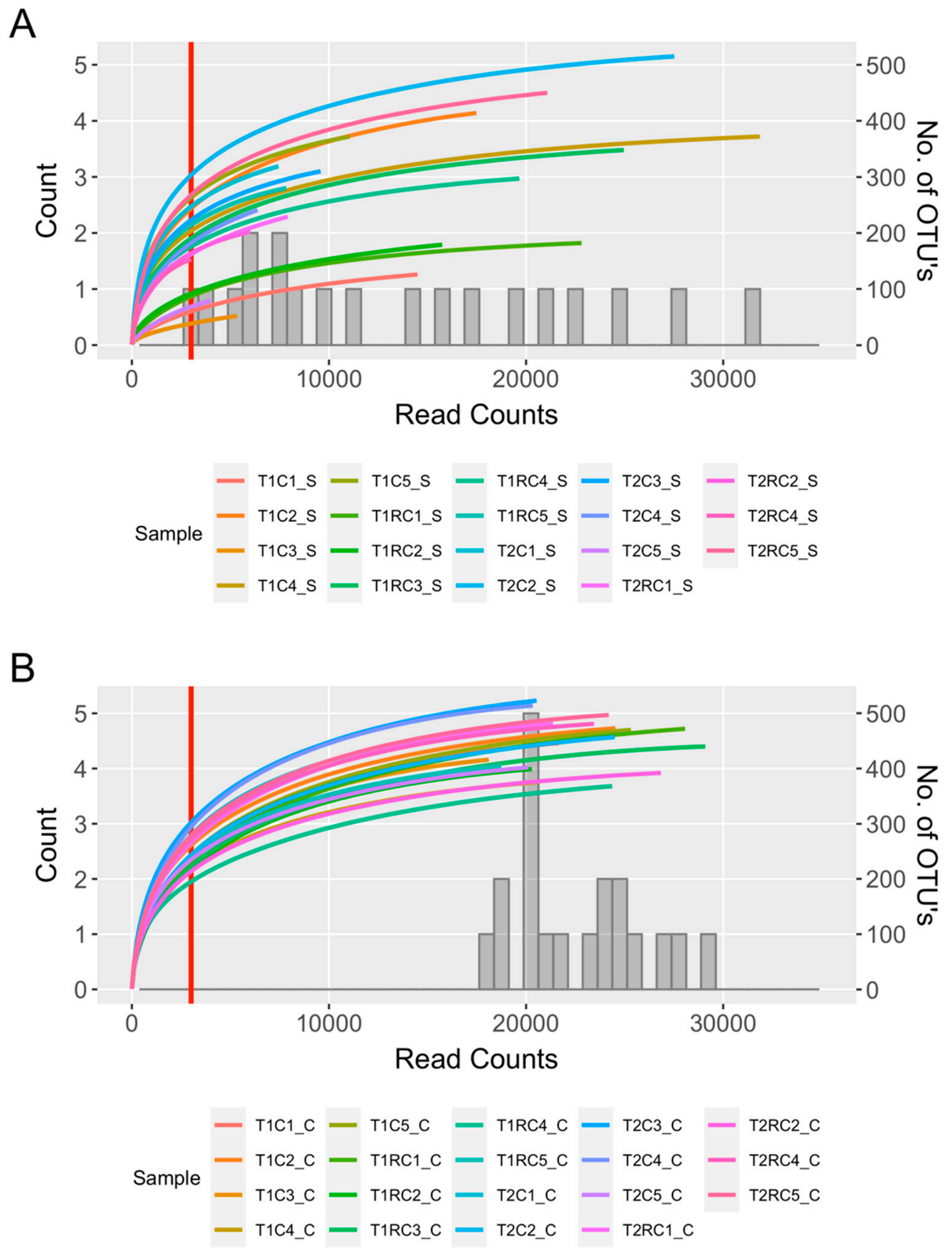

Figure S1. (A) Rarefaction curves for the cloacal swab samples, where curve color designates each of the nineteen individual samples. The red vertical line at 3005 indicates the minimum read count threshold necessary for further analysis. The histogram in the background displays the number of rarefaction curves (samples) that

terminate at a specific read count depth. **(B)** Rarefaction curves for the cecal content samples, where curve color designates each of the nineteen individual samples. The red vertical line at 3005 indicates the minimum read count threshold necessary for further analysis. The histogram in the background displays the number of rarefaction curves (samples) that terminate at a specific read count depth.

Figure S2. QQ-Plots for Cloacal swabs and Cecal Content

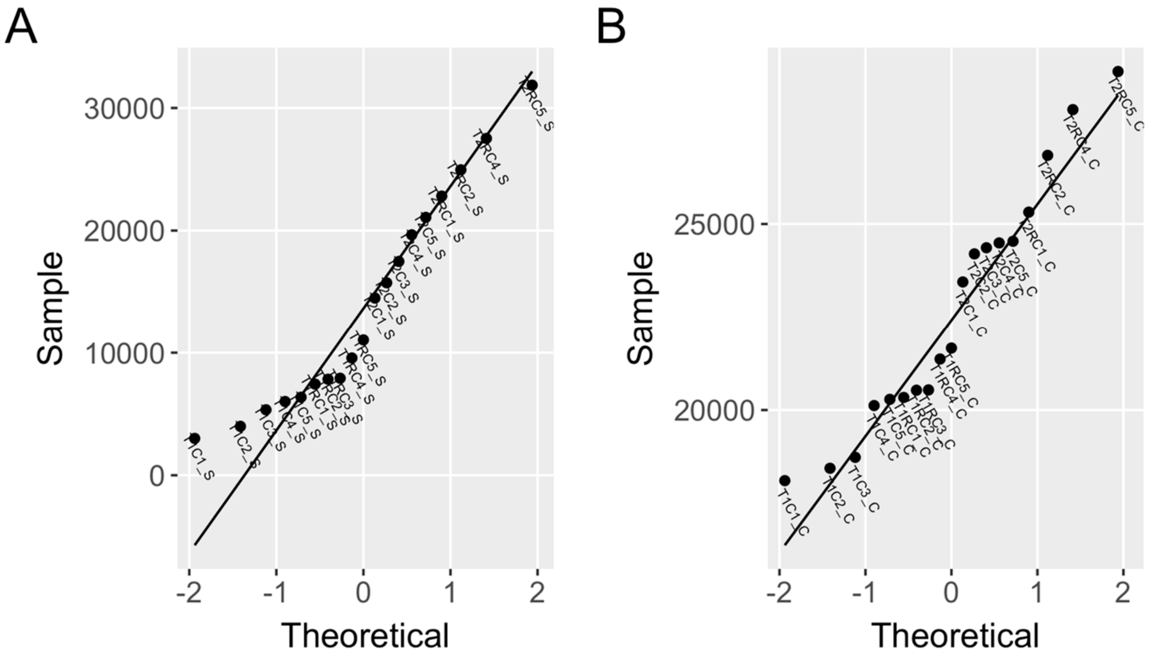

Figure S2. (A) Q-Q plots of the Goods Coverage Index values for nineteen Cloacal Swab Samples. **(B)** Q-Q plots of the Goods Coverage Index values for nineteen Cecal Content Samples.

Table S1. Raw sequencing data for the nineteen Cloacal Swab samples.

Sample	Reads	Good's Coverage	Method	Treatment
T1C1_S	14,493	99.70%	Cloacal_Swab	Treatment_1
T1C2_S	17,480	99.57%	Cloacal_Swab	Treatment_1
T1C3_S	5,342	99.49%	Cloacal_Swab	Treatment_1
T1C4_S	31,862	99.86%	Cloacal_Swab	Treatment_1
T1C5_S	11,043	99.34%	Cloacal_Swab	Treatment_1
T1RC1_S	22,812	99.86%	Cloacal_Swab	Treatment_1
T1RC2_S	15,741	99.66%	Cloacal_Swab	Treatment_1
T1RC3_S	24,955	99.79%	Cloacal_Swab	Treatment_1
T1RC4_S	19,655	99.75%	Cloacal_Swab	Treatment_1
T1RC5_S	7,832	99.27%	Cloacal_Swab	Treatment_1
T2C1_S	7,434	99.00%	Cloacal_Swab	Treatment_2
T2C2_S	27,516	99.77%	Cloacal_Swab	Treatment_2
T2C3_S	9,568	99.29%	Cloacal_Swab	Treatment_2
T2C4_S	6,369	98.70%	Cloacal_Swab	Treatment_2
T2C5_S	3,988	99.07%	Cloacal_Swab	Treatment_2
T2RC1_S	7,912	99.08%	Cloacal_Swab	Treatment_2
T2RC2_S	6,013	99.22%	Cloacal_Swab	Treatment_2
T2RC4_S	3,005	98.74%	Cloacal_Swab	Treatment_2
T2RC5_S	21,076	99.64%	Cloacal_Swab	Treatment_2

Table S1. Summary of the sequencing data for the nineteen Cloacal Swab samples that passed the quality filtering parameter after executing the MOTHUR pipeline. Sample names are listed in the first column, with the sequencing reads, Good's coverage percentage, sampling method, and treatment for the specified sample in the adjacent columns.

Table S2. Raw sequencing data for the nineteen Cecal Content samples.

Sample	Reads	Good's Coverage	Method	Treatment
T1C1_C	21,662	99.71%	Cecal_Content	Treatment_1
T1C2_C	24,537	99.72%	Cecal_Content	Treatment_1
T1C3_C	18,106	99.64%	Cecal_Content	Treatment_1
T1C4_C	20,538	99.72%	Cecal_Content	Treatment_1
T1C5_C	25,318	99.71%	Cecal_Content	Treatment_1
T1RC1_C	28,066	99.75%	Cecal_Content	Treatment_1
T1RC2_C	20,286	99.72%	Cecal_Content	Treatment_1
T1RC3_C	29,088	99.86%	Cecal_Content	Treatment_1
T1RC4_C	24,363	99.73%	Cecal_Content	Treatment_1
T1RC5_C	18,731	99.65%	Cecal_Content	Treatment_1
T2C1_C	18,438	99.68%	Cecal_Content	Treatment_2
T2C2_C	24,493	99.71%	Cecal_Content	Treatment_2
T2C3_C	20,530	99.61%	Cecal_Content	Treatment_2
T2C4_C	20,337	99.73%	Cecal_Content	Treatment_2
T2C5_C	20,119	99.69%	Cecal_Content	Treatment_2
T2RC1_C	21,367	99.71%	Cecal_Content	Treatment_2
T2RC2_C	26,840	99.83%	Cecal_Content	Treatment_2
T2RC4_C	23,446	99.77%	Cecal_Content	Treatment_2
T2RC5_C	24,198	99.77%	Cecal_Content	Treatment_2

Table S1. Summary of the sequencing data for the nineteen Cecal Content samples that passed the quality filtering parameter after executing the MOTHUR pipeline. Sample names are listed in the first column, with the sequencing reads, Good's coverage percentage, sampling method, and treatment for the specified sample in the adjacent columns.
